# 3-Deoxysappanchalcone Inhibits Skin Cancer Proliferation by Regulating T-Lymphokine-Activated Killer Cell-Originated Protein Kinase *in vitro* and *in vivo*

**DOI:** 10.3389/fcell.2021.638174

**Published:** 2021-03-25

**Authors:** Xiaorong Fu, Ran Zhao, Goo Yoon, Jung-Hyun Shim, Bu Young Choi, Fanxiang Yin, Beibei Xu, Kyle Vaughn Laster, Kangdong Liu, Zigang Dong, Mee-Hyun Lee

**Affiliations:** ^1^Department of Pathophysiology, School of Basic Medical Sciences, College of Medicine, Zhengzhou University, Zhengzhou, China; ^2^China-US (Henan) Hormel Cancer Institute, Zhengzhou, China; ^3^Department of Pharmacy, College of Pharmacy, Mokpo National University, Muan, South Korea; ^4^Department of Pharmaceutical Science and Engineering, School of Convergence Bioscience and Technology, Seowon University, Cheongju, South Korea; ^5^Department of Translational Medicine Center, The First Affiliated Hospital of Zhengzhou University, Zhengzhou, China; ^6^College of Korean Medicine, Dongshin University, Naju, South Korea

**Keywords:** skin cancer, solar sinulated light, T-LAK cell-originated protein kinase, 3-deoxysappanchalcone, cancer growth, skin hyperplasia

## Abstract

**Background:**

Skin cancer is one of the most commonly diagnosed cancers worldwide. The 5-year survival rate of the most aggressive late-stage skin cancer ranges between 20 and 30%. Thus, the discovery and investigation of novel target therapeutic agents that can effectively treat skin cancer is of the utmost importance. The T-lymphokine-activated killer cell-originated protein kinase (TOPK), which belongs to the serine-threonine kinase class of the mitogen-activated protein kinase kinase (MAPKK) family, is highly expressed and activated in skin cancer. The present study investigates the role of 3-deoxysappanchalcone (3-DSC), a plant-derived functional TOPK inhibitor, in suppressing skin cancer cell growth.

**Purpose:**

In the context of skin cancer prevention and therapy, we clarify the effect and mechanism of 3-DSC on different types of skin cancer and solar-simulated light (SSL)-induced skin hyperplasia.

**Methods:**

In an *in vitro* study, western blotting and *in vitro* kinase assays were utilized to determine the protein expression of TOPK and its activity, respectively. Pull-down assay with 3-DSC and TOPK (wild-type and T42A/N172 mutation) was performed to confirm the direct interaction between T42A/N172 amino acid sites of TOPK and 3-DSC. Cell proliferation and anchorage-independent cell growth assays were utilized to determine the effect of 3-DSC on cell growth. In an *in vivo* study, the thickness of skin and tumor size were measured in the acute SSL-induced inflammation mouse model or SK-MEL-2 cell-derived xenografts mouse model treated with 3-DSC. Immunohistochemistry analysis of tumors isolated from SK-MEL-2 cell-derived xenografts was performed to determine whether cell-based results observed upon 3-DSC treatment could be recapitulated *in vivo*.

**Results:**

3-DSC is able to inhibit cell proliferation in skin cancer cells in an anchorage-dependent and anchorage-independent manner by regulation of TOPK and its related signaling pathway *in vitro*. We also found that application of 3-DSC reduced acute SSL-induced murine skin hyperplasia. Additionally, we observed that 3-DSC decreased SK-MEL-2 cell-derived xenograft tumor growth through attenuating phosphorylation of TOPK and its downstream effectors including ERK, RSK, and c-Jun.

**Conclusions:**

Our results suggest that 3-DSC may function in a chemopreventive and chemotherapeutic capacity by protecting against UV-induced skin hyperplasia and inhibiting tumor cell growth by attenuating TOPK signaling, respectively.

## Introduction

Skin cancer is one of the most commonly diagnosed cancers worldwide and poses a huge financial burden to society (Linares et al., 2015). Skin cancer can be classified as basal cell carcinoma (BCC), squamous cell carcinoma (SCC), or melanoma. Excess solar ultraviolet (SUV) radiation is the dominant risk factor for skin cancer (Ratushny et al., 2012; Gao et al., 2017). It has been suggested that acute solar-simulated light (SSL) irradiation enhances T-lymphokine-activated killer cell-originated protein kinase (TOPK) expression and induces inflammation in SKH1 mice dorsal skin (Xue et al., 2017; Roh et al., 2018). Therefore, targeting TOPK may be a promising strategy for skin cancer prevention and treatment (Gao et al., 2017; Roh et al., 2018).

TOPK, a serine-threonine kinase, is a member of the mitogen-activated protein kinase kinase (MAPKK) family (Dougherty et al., 2005) and exists at the same hierarchical level of MEK in the MAPK signaling axis. Mutations in the MAPK signaling cascade are pronounced in many types of cancer (Braicu et al., 2019). Previous research has established that TOPK can induce cancer cell proliferation and is highly expressed in numerous types of cancer including lung adenocarcinoma (Lei et al., 2013), ovarian cancer (Ikeda et al., 2016), gastric carcinoma (Ohashi et al., 2017), breast cancer (Park et al., 2006), nasopharyngeal carcinoma (Wang et al., 2016), and colon cancer (Zhu et al., 2007). TOPK regulates a wide range of tumor processes such as tumor growth, invasion (Lee et al., 2020), development (Hu et al., 2010), and resistance (Herbert et al., 2018). As a MAPKK member in MAPK signal pathway, TOPK inhibitors may be utilized to treat MEK-resistant patients or in the form of target combination therapy. Until now, there have been no clinical trials to investigate the therapeutic potential of TOPK inhibitors. As such, active investigation of TOPK inhibitors may provide a means of reducing cancer growth across many different types of cancer. To date, the efficacy of several TOPK inhibitors have been investigated *in vitro* and *in vivo*. However, variable degrees of cytotoxicity have been observed, which may clinically translate to diminished patient quality of life. Therefore, there is an unmet need for TOPK inhibitors with minimal toxicity.

In the present manuscript, we characterize the role 3-deoxysappanchalcone (3-DSC), a natural compound derived from *Caesalpinia sappan* L., which plays as a novel TOPK inhibitor in skin carcinogenesis and cancer. We report that 3-DSC effectively inhibited skin cancer cell growth by directly attenuating TOPK signaling *in vitro*. Additionally, an *in vivo* study showed that 3-DSC reduced SSL-induced skin hyperplasia and tumor growth in a SK-MEL-2 cell-derived xenograft mouse model. Our observations suggest that 3-DSC functions as an effective anti-cancer prophylactic and chemotherapeutic reagent that may be used to clinically treat skin cancer.

## Materials and Methods

### Reagents

3-DSC (purity ≥ 95%, C_16_H_14_O_4_, CAS No. 112408-67-0), a natural compound isolated from the plant of *Caesalpinia sappan* L. (Leguminosae), was purchased from ChemFaces (Wuhan, Hubei, China). Active TOPK kinase (100 ng, Cat#T14-10G), kinase dilution buffer (Cat#K01-09-20), and 10 mM ATP (Cat#A50-09) were purchased from SignalChem Biotech Inc. (Richmond, BC, Canada). Recombinant human myelin basic protein (MBP) (Cat#M42-51N) was purchased from Sigma-Aldrich (St. Louis, MO, United States). Matrigel was purchased from Corning (Tewksbury, MA, United States). Protease inhibitor cocktail (100X, HY-K0010) and phosphatase inhibitor cocktail II (100X, HY-K0022) were purchased from MedChemExpress (Monmouth Junction, NJ, United States). Antibodies to detect rabbit anti-cleaved PARP (1:1,000, Cat#5625), rabbit anti-PARP (1:1,000, Cat#9542), rabbit anti-caspase 3 (1:1,000, Cat#9662), rabbit anti-caspase 7 (1:1,000, Cat#9492), rabbit anti-cleaved caspase 3 (1:1,000,Cat#9664), rabbit anti-cleaved caspase 7 (Asp 198, 1:1,000, Cat#8438), rabbit anti-cyclin B1 (1:1,000, Cat#12231), rabbit anti-phosphorylated ERK (Thr202/Tyr204) (1:1,000, Cat#4370), rabbit anti-phosphorylated TOPK (Thr9) (1:1,000, Cat#4941), rabbit anti-phosphorylated c-Jun (Ser63) (1:1,000, Cat#2361), rabbit anti-phosphorylated p90RSK (Ser380) (1:1,000, Cat#11989), rabbit anti-ERK1/2 (p44/p42 MAPK) (1:1,000, Cat#4695), rabbit anti-TOPK (1:1,000, Cat#4942), rabbit anti-c-Jun (1:1,000, Cat#9165), and rabbit anti-RSK2 (1:1,000, Cat#5528) were purchased from Cell Signaling Technology (Beverly, MA, United States). Antibodies to detect mouse anti-phosphoserine (1:1,000, Cat#ab9332), rabbit anti-phosphothreonine (1:1,000, Cat#ab9337), and rabbit anti-Ki67 (1:200, Cat#ab16667) were purchased from Abcam Inc. (Cambridge, United Kingdom). Xfect Transfection Reagent was purchased from TaKaRa Biomedical Technology Beijing Co., Ltd. (Beijing, China).

### Cell Lines and Cell Culture

Skin normal cell JB6 CI 41-5a (JB6); HaCaT cells; Normal Human Dermal Fibroblasts (NHDF); and skin cancer cell lines SK-MEL-2, SK-MEL-28, A375, and A431 were purchased from American Type Culture Collection (ATCC, Manassas, VA, United States). Cells were cultured in Minimum Essential Medium (MEM)/Earle’s Balanced Salt Solution (EBSS) (SK-MEL-2), MEM (SK-MEL-28), and Dulbecco’s Modified Eagle’s medium (DMEM) (A375 and A431) supplemented with 10% fetal bovine serum and penicillin/streptomycin (1×). Normal skin cell line JB6, HaCaT, and NHDF were cultured in MEM and DMEM medium supplemented with 5 or 10% fetal bovine serum and penicillin/streptomycin (1×). All the cells used in this study were authenticated through STR profiling and maintained at 37°C in a 5% CO_2_ humidified incubator.

### Western Blot Analysis

Cells were rinsed with phosphate-buffered saline (PBS) before being scraped and lysed in radioimmunoprecipitation assay (RIPA) buffer supplemented with protease inhibitor cocktail and phosphatase inhibitor cocktail II. After centrifugation at 14,000 rpm for 15 min, the supernatant fractions were harvested as the total cellular protein extracts. The protein concentration was determined using a protein assay kit (Solarbio Life Science, Beijing, China). The total cellular protein extracts were separated by sodium dodecyl sulfate polyacrylamide gel electrophoresis (SDS-PAGE) and transferred to polyvinylidene fluoride membranes in 20 mM Tris–HCl (pH 8.0), 150 mM glycine, and 20% (*v*/*v*) methanol. Membranes were blocked with 5% nonfat dry milk in 1xPBS containing 0.05% Tween-20 (PBS-T) and incubated with antibodies against p-TOPK, TOPK, p-ERK1/2, ERK1/2, p-RSK2, RSK2, p-c-Jun, total c-Jun, PARP, caspase 3, caspase 7, cleaved PARP, cleaved caspase 3, cleaved caspase 7, or β-actin at 4°C, overnight. Blots were washed three times with 1xPBS-T buffer, followed by incubation with the appropriate horseradish peroxidase-linked immunoglobulin G (IgG). The specific proteins in the blots were visualized using an enhanced chemiluminescence detection reagent and the Amersham Imager 600 (GE Healthcare life Science, Pittsburgh, PA, United States).

### The Cancer Genome Atlas Database Analysis

Gene expression and patient clinical data of The Cancer Genome Atlas-Skin Cutaneous Melanoma (TCGA-SKCM) cohort was downloaded using TCGA GDAC Firehose [Broad Institute TCGA Data Analysis Center (2016): Firehose 2016/01/28, Broad Institute of MIT and Harvard; doi: 10.7908/C11G0KM9). RNA-seq data detailing TOPK expression in normal skin samples unexposed to sun were downloaded from the GTEx database. RSEM values were transformed to transcript per million (TPM) values by multiplying scaled estimates by 1E7. TOPK gene expression was plotted in TPM units with respect to features such as tumor stage, patient age, race, weight, etc., by cross-querying clinical data. Statistical significance (*p* < 0.05) of gene expression was assessed using a Wilcoxon rank-sum test. Whisker charts illustrating the median, upper, and lower quantiles of TPM expression values were generated using Mathematica 12.

### *In vitro* Kinase Assay

Active TOPK kinase (100 ng), recombinant human myelin basic protein (MBP substrate, 200 ng), kinase assay buffer, and 100 μM ATP were incubated at 30°C for 30 min in the presence or absence of 3-DSC. To stop the reaction, 5 μl of 6μ loading buffer was then added. The protein mixture was loaded into a 12% SDS-PAGE gel and run as a western blot to quantify p-serine/threonine (1:1,000) expression.

### *In vitro* Pull-Down Assay

Skin cancer cells, A431, A375, SK-MEL-2, and SK-MEL-28, were cultured in complete growth medium until an optimal confluence of 70% was obtained. Cells were rinsed with PBS before being scraped and lysed in RIPA buffer. The individual cell lysates were incubated with Sepharose 4B beads or 3-DSC-Sepharose 4B beads in reaction buffer (50 mM Tris–HCl, pH 7.5, 5 mM EDTA, 150 mM NaCl, 1 mM DTT, 0.01% NP-40, 0.2 mM PMSF, and 20× protease inhibitor). After incubation with gentle rocking overnight at 4°C, the beads were washed three times with washing buffer (50 mM Tris–HCl, pH 7.5, 5 mM EDTA, 150 mM NaCl, 1 mM DTT, 0.01% NP-40, and 0.2 mM PMSF), and binding was visualized by western blotting against TOPK antibody. For the ATP competitive binding assay, active TOPK was incubated with 3-DSC-Sepharose 4B beads and vehicle, 10, 100, or 1,000 μM ATP, following the procedure described above for the binding assay.

### Construction and Expression of TOPK Wild Type and Mutants

*pcDNA4.1-HisC-TOPK* encoding recombinant plasmid was used to construct TOPK-T42A by site mutagenesis with Hieff MutTM Site-Directed Mutagenesis Kit (Yeasen Biotech Co., Ltd, Shanghai, China). The forward primer of TOPK T42A used was as follows: 5′-TTGGCTTTGGTGCTGGGGTA-3′. The reverse primer of TOPK T42A used was as follows: 5′-GCAAGATGGTTATGAAGGCAGG-3′. Annealing temperature was tested to be 57°C. Then, TOPK-T42A or *pcDNA4.1-HisC-TOPK* was applied to get TOPK N172A + T42A or TOPK N172A by overlap PCR assay. The primer sequences here were as follows:

TOPK-N172A-forward-1: 5′-TTGAATTCATGGAAGGGAT CAGTAATT TCAAG-3′;
TOPK-N172A-reverse-1: 5′- AAATCGCCTTTAATTACAAC AGCTGAAGAC-3′;
TOPK-N172A-forward-2: 5′-CTTCATGGAGACATAAAGT CTTCAGCTGTTGT-3′;
TOPK-N172A-reverse-2: 5′-TTGCGGCCGCCTAGACATCT GTTTCCAGAGC-3′.

Annealing temperature here was 54°C. The recombinant plasmids were transformed to *E. coli* DH5μ and colonies were sent to analyze the sequences. TOPK-T42A, TOPK-N172A, and TOPK-T42A-N172A plasmids were extracted by AxyPrep^TM^ Plasmid Midiprep Kit (Corning, Tewksbury, MA, United States). TOPK wild-type or mutants were transfected to HEK-293T cells by Xfect^TM^ Transfection Reagent, and cells were rinsed with PBS before being scraped and lysed in RIPA buffer and incubated with antibodies against p-TOPK, TOPK, p-ERK1/2, ERK1/2, p-RSK2, RSK2, p-c-Jun, total c-Jun, or β-actin.

### Acute SSL Exposure to 3-DSC-Treated Mouse

Five–six weeks SKH1 male mice were maintained under specific pathogen-free (SPF) conditions. Mice with similar body weight (18–20 g) were divided into four groups as follows: (1) acetone (vehicle), (2) acetone + SSL, (3) 500 nmol 3-DSC + SSL, and (4) 1,000 nmol 3-DSC + SSL. Acetone or 3-DSC were topically applied onto the dorsal skin of SKH1 mice. One hour later, 149 kJ/m^2^ UVA and 7.2 kJ/m^2^ SSL were applied to the dorsal skin of the mice. After treatment, 24 h later, the irradiated dorsal skin tissue (about 2 cm^2^) was harvested. Hematoxylin and eosin (H&E) staining and immunohistochemistry were performed on the tissues using p-PBK/TOPK (Thr9), p-ERK1/2 (Thr202/Tyr204), p-c-Jun (Ser63), and p-p90RSK (Ser380) antibodies.

### Cell Proliferation Assay

The cells (A431, A375, SK-MEL-2, and SK-MEL-28) were seeded (2 × 10^3^ cells per well for) in 96-well plates and incubated for 24 h and then treated with different doses of 3-DSC or vehicle. After incubation for 24, 48, 72, or 96 h, cell proliferation was measured by MTT assay.

### Anchorage-Independent Cell Transformation Assay

The normal cells (JB6 CI 41-5a or HaCaT) or the skin cancer cells (A431, A375, SK-MEL-2, and SK-MEL-28) suspended in complete Basal Medium Eagle growth medium were seeded (8,000 cells/well) into 0.3% agar with or without epidermal growth factor (EGF) (10 ng/ml) and 3-DSC (0, 5, 10, and 20 μM) over a base layer of 0.6% agar containing the same concentration of EGF and 3-DSC. The cultures were maintained at 37°C in a 5% CO_2_ incubator for 3 weeks. Colonies were visualized using an inverted microscope and counted using the Image−Pro Plus software (v.6) program (Media Cybernetics, Rockville, MD, United States).

### Knock-Down of TOPK Expression

Each viral vector and packaging vectors (pMD2G, psPAX2, and shTOPK#1 and 2) were transfected using the Xfect Transfection Reagent into Lenti-X-293T cells. The viral particles were harvested by filtration using a 0.22-μm filter and then stored at −20°C. The cultured SK-MEL-2 and A375 skin cancer cells were infected with virus particles and 8 μg/ml polybrene (Millipore, Billerica, MA, United States, Cat#TR-1003) for 24 h. Then, the cells were selected with puromycin for 24 h, and the selected cells were used for anchorage-independent cell growth assay and western blot analysis.

### Cell Cycle and Apoptosis Analysis

The cells (2 × 10^5^ cells) were seeded in 60-mm dishes and treated with 0, 5, 10, or 20 μM 3-DSC for 48 h. For cell cycle analysis, the cells were fixed in 70% ethanol and stored at −20°C for 24 h. After staining with annexin-V for apoptosis or propidium iodide for cell cycle assessment, the cells were analyzed using a BD FACSCalibur Flow Cytometer (BD Biosciences, San Jose, CA, United States).

### Cell-Derived Xenograft Mouse Model

All animal experiments were approved by the Ethics Committee of China-US (Henan) Hormel Cancer Institute. Nu/Nu mice (5–6 weeks old) were maintained under standard SPF mice room. We injected 5 × 10^6^ SK-MEL-2 cells per mouse into near the right hind leg for inducing cell-derived xenograft tumors. After 1 week, the cell-derived xenograft (CDX) tumor’s average size growth was measured to 100 mm^3^, and the tumors were randomly separated into three groups (vehicle, 10, or 20 mg/kg; 10 mice each group). Then mice were treated with 3-DSC (10 or 20 mg/kg) or vehicle by intraperitoneal injection every day throughout the whole experiment. Mice were euthanized after 43 days; the tumor, blood, spleen, and the liver were collected.

### Immunohistochemical Analysis

Tumors were collected from the mice and fixed, and paraffin-embedded sections (3 μm) were prepared for hematoxylin and eosin (H&E) staining and immunohistochemical (IHC) analysis. After antigen unmasking, the sections were blocked with 5% goat serum and incubated at 4°C overnight with antibodies Ki-67 (1:200), p-TOPK (1:200), p-ERK1/2 (1:100), p-RSK (1:100), and p-c-Jun (1:100). After incubation with a rabbit secondary antibody, DAB (3,3-diaminobenzidine) staining was used following the manufacturer’s instructions to visualize the protein targets. Sectioned tissues were counterstained with hematoxylin, dehydrated through a graded series of alcohol into xylene, and mounted under glass coverslips. Images were obtained using Olympus Imaging BX43 (Southborough, MA, United States). The fluorescence intensity was quantified using Image-Pro Plus software (version 6.0).

### Statistical Analysis

All other quantitative results are expressed as mean values ± SD. Data was analyzed by one-way ANOVA or Student’s *t* test. A value of *p* < 0.05, *p* < 0.001 was indicated as “*”, “***” for statistical significance.

## Results

### 3-DSC Inhibited TOPK Kinase Activity by Binding at the Sites of Thr42 and Asn172

**FIGURE 1:**
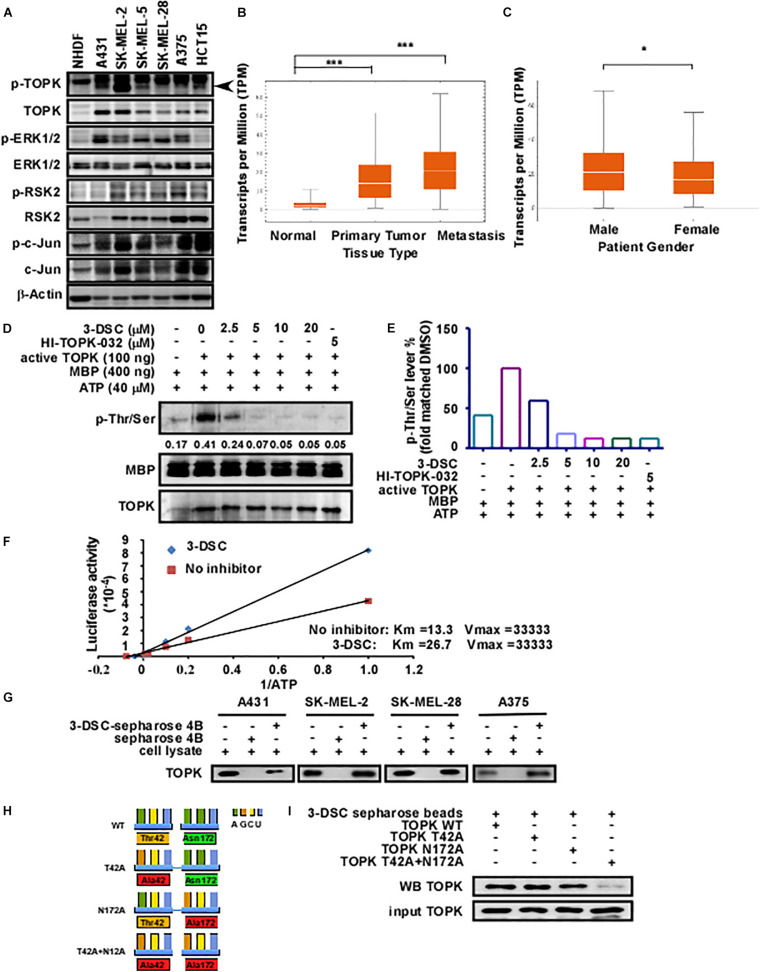
3-Deoxysappanchalcone (3-DSC) inhibits T-LAK cell-originated protein kinase (TOPK) kinase activity by binding to Thr42 and Asn172. **(A)** The protein expression levels of TOPK and its downstream protein effectors in Normal Human Dermal Fibroblast (NHDF) and skin cancer cell lines (A431, SK-MEL2, SK-MEL-28, and A375) and colon cancer cell line HCT15 (positive control). **(B,C)** The expression levels of TOPK dependent on the tumor type (primary melanoma patients, metastatic melanoma patient, and normal person) or gender from The Cancer Genome Atlas-Skin Cutaneous Melanoma (TCGA-SKCM) cohort database. TOPK transcript expression was analyzed according to tissue type according to transcript per million (TPM) units. **(D)** Effect of 3-DSC on TOPK phosphorylation. Kinase assay was performed with active TOPK (100 ng), 3-DSC (2.5, 5, 10, or 20 μM), HI-TOPK-032 (5 μM), myelin basic protein (MBP) (400 ng), and ATP (40 μM). MBP is the substrate of TOPK kinase; p-Thr/Ser levels are an indicator of TOPK activity. The band intensity of p-Thr/Ser is significantly reduced when the assay was performed with inclusion of 2.5 μM 3-DSC. **(E)** Band quantification results of **(D)**. **(F)** Kinetic analysis of 3-DSC whether competing ATP with TOPK substrate. Km and Vm values of TOPK enzyme incubated with or without 3-DSC. **(G)** Binding affinity of 3-DSC to TOPK was assessed in four skin cancer cells. 3-DSC-Sepharose 4B or Sepharose 4B were incubated with cell lysates. Protein was then eluted from the beads and separated by SDS-PAGE. **(H)** Diagram illustrating the mutation of residues previously predicted to be important in facilitating the interaction between TOPK and 3-DSC. TOPK was mutated at the T42A, N172A, and T42A residues based upon computer docking analysis. **(I)** *In vitro* pull-down assay with TOPK WT, TOPK T42A, TOPK N172A, and TOPK T42A + N172A. Double mutation significantly attenuated binding of 3-DSC with TOPK. *p < 0.05, ***p < 0.001.

PBK/TOPK is highly expressed in several types of cancer including lung adenocarcinoma (Xiao et al., 2019), ovarian cancer (Ikeda et al., 2016), gastric carcinoma (Ohashi et al., 2017), breast cancer (Park et al., 2006), nasopharyngeal carcinoma (Wang et al., 2016), and colon cancer (Zhu et al., 2007). To determine protein expression levels of TOPK in the context of skin cancer, we quantified protein expression in several skin cancer lines by western blot. Our results showed that TOPK was highly expressed in skin cancer cells relative to the normal human dermal fibroblast cell line, with the HCT-15 colon cancer cell line serving as a positive control (Figure 1A). We next queried the SKCM cohort of the TCGA database to determine whether TOPK expression is associated with patient features and cancer staging. As RNA-seq data from normal skin samples is under-represented within the TCGA-SKCM cohort, we supplemented the TCGA data with RNA-seq data from the GTEx database detailing TOPK expression in normal skin that was unexposed to sunlight. The results confirm that TOPK is significantly over-expressed in primary and metastatic melanoma tumors compared to normal tissues. The results also show that TOPK expression is significantly increased in the case of metastatic melanoma compared to that in primary tumors (Figure 1B). Additionally, there appear to be gender-specific differences in the expression of TOPK in tumor tissues, with the TOPK profile in tumors derived from male patients exhibiting increased expression (Figure 1C). We observed that transcript expression does not appreciably change across race (Supplementary Figure 1A), age (Supplementary Figure 1B), and stage (Supplementary Figure 1C). Thus, based upon the aforementioned observations, we hypothesized that TOPK could play a role in the establishment or maintenance of skin cancer and that inhibition of TOPK may be useful in treating skin cancer. We recently reported that 3-DSC could suppress cell growth by directly binding to TOPK in colon cancer (Zhao et al., 2019). As TOPK is over-expressed in skin cancer, we sought to determine whether we could achieve comparable targeting efficacy in different types of skin cancer. Thus, we measured the effect of 3-DSC on TOPK activity by performing kinase assay using lysates derived from skin cancer cells (Figures 1D,E) and ATP competitive kinase assays with an increasing Km value from 13.3 to 26.7 (Figure 1F). The results indicate that 3-DSC inhibited TOPK activity in an ATP-competitive manner (Figures 1D–F). *In vitro* pull-down results showed that 3-DSC could directly bind with TOPK in different skin cancer cell lines (Figure 1G). Our previous work utilized a computer docking model to predict that 3-DSC binds with TOPK at the Thr42 and Asn172 residues. However, this prediction was not experimentally validated. Therefore, we established TOPK plasmid constructs harboring mutations at the predicted binding sites, denoted as TOPK T42A, TOPK N172A, and TOPK T42A + N172A (Figure 1H), to verify the computer model predictions by pull-down assay. Our results suggest that the prediction made by the computer docking model was valid, as the TOPK double mutant (T42A + N172A) greatly reduced binding affinity between 3-DSC and TOPK (Figure 1I).

### 3-DSC Decreased Skin Epidermal Thickness Caused by SSL Exposure

**FIGURE 2:**
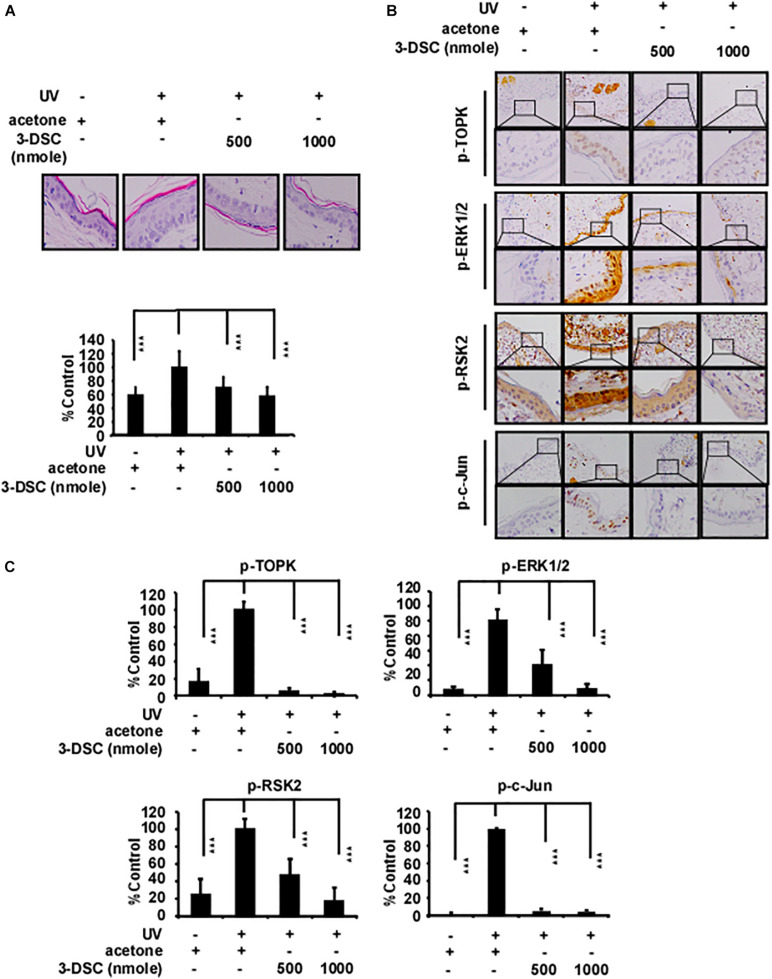
3-DSC decreases skin epidermal thickening caused by solar ultraviolet (SUV) exposure by targeting TOPK. **(A)** Representative hematoxylin and eosin (H&E) staining results of mice dorsal skin treated with or without 3-DSC prior to SUV exposure. Each group consisted of 8–10 mice. Tissue thickness was measured using a microscope ruler. Data are shown as mean ± SD values. **(B)** Protein expressions of p-TOPK, p-ERK, p-RSK, and p-c-Jun were detected by immunohistochemical (IHC) analysis. **(C)** Quantification of the IHC results in panel **(B)**. Data are shown as mean ± SD values. ***p < 0.001.

Actinic keratosis (AK) is characterized by accretive atypical keratinocytes, which results in abnormally thickened skin. AK is a precursor to cutaneous squamous cell carcinoma (cSCC) (Ratushny et al., 2012). Acute solar ultraviolet (SUV), a major contributor to the emergence of AK, increases TOPK protein levels in human skin tissue (Roh et al., 2018). Therefore, we investigated if 3-DSC can be utilized to prevent skin thickening through targeting TOPK. We applied two doses of 3-DSC onto SKH1 hairless mice before administering SSL and harvesting tissue for H&E staining and IHC analysis. We observed that application of 3-DSC decreased epidermal thickening in addition to decreasing TOPK, ERK, RSK, and c-Jun protein phosphorylation levels (Figures 2A–C). This finding strongly suggests that 3-DSC may possess chemoprevention potential with respect to SSL-induced damage by directly attenuating TOPK and its downstream effectors.

### 3-DSC Inhibited Skin Cancer Cell Proliferation

**FIGURE 3:**
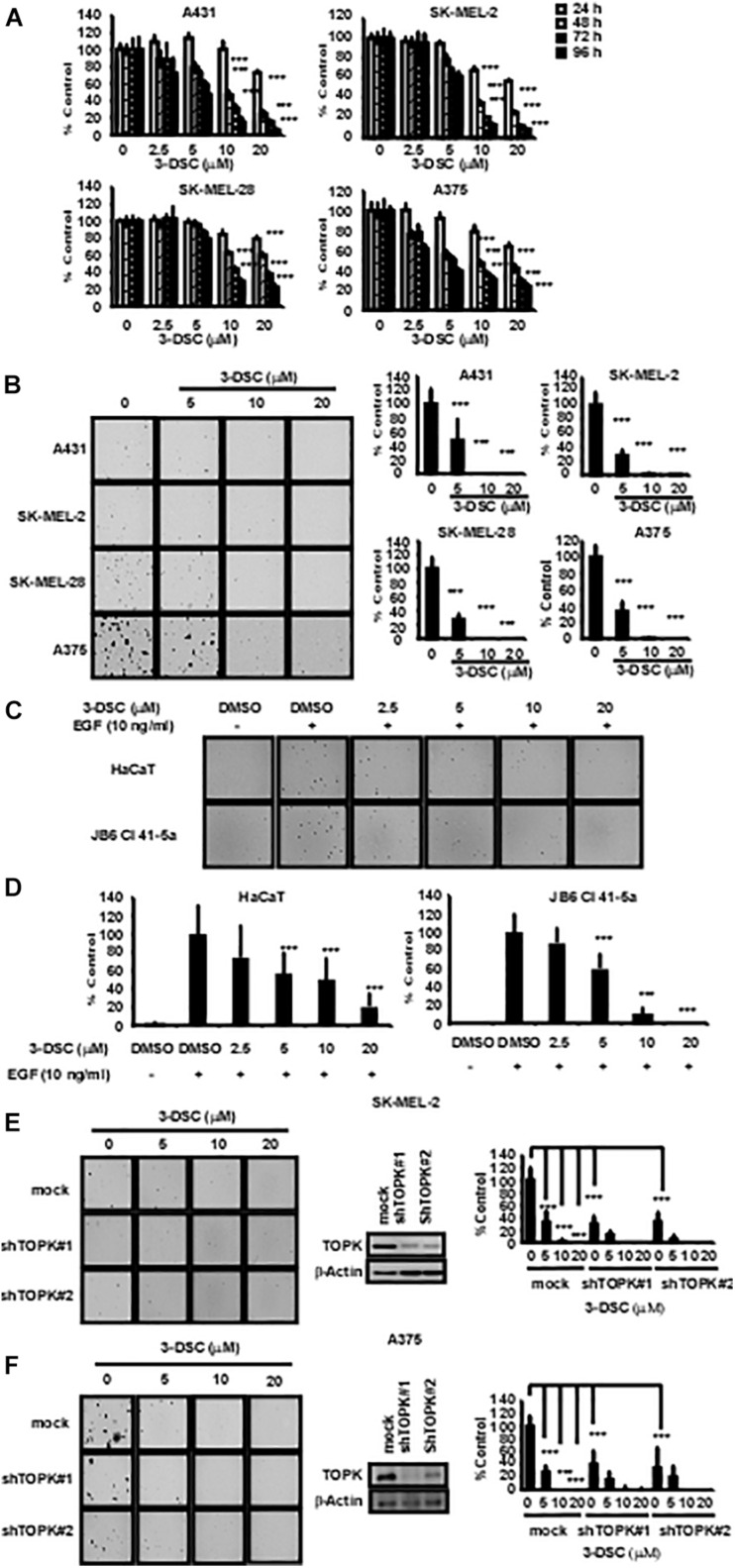
3-DSC inhibits anchorage-dependent and independent growth in skin cancer cell lines. **(A)** The inhibition effects of different concentrations of 3-DSC in cell proliferation of A431, SK-MEL-2, SK-MEL-28, and A375 cells. Skin cancer cells were incubated with 3-DSC for 24, 48, 72, and 96 h and growth was detected by MTT assay. Data shown as mean ± SD values. **(B)** Representative images of anchorage-independent soft agar growth assay using skin cancer cell lines incubated with various concentrations of 3-DSC. **(C)** Representative images showing the effect of 3-DSC on anchorage-independent growth of EGF-induced transformation of normal skin cells. **(D)** Quantification of colony numbers shown in panel **(C)**. Data are shown as mean ± SD values. **(E)** SK-MEL-2 cell anchorage-independent colony growth after knocking down TOPK and treating with various concentrations of 3-DSC. Colony number is quantified in the right side of the panel. **(F)** A375 cell anchorage-independent colony growth after knocking down TOPK and treating with various concentrations of 3-DSC. Colony number is quantified in the right side of the panel. ***p < 0.001.

To determine the toxicity of 3-DSC, we performed an MTT assay over a concentration gradient of 3-DSC ranging between 0 and 20 μM using NHDF cells. The results indicated that 3-DSC showed minimal toxicity in NHDF cells at the tested concentrations (Supplementary Figure 2A). Based on the results of the toxicity assay, we then treated A431, SK-MEL-2, SK-MEL-28, and A375 skin cancer cell lines with 3-DSC over a range of 0–20 μM. After incubating for 24, 48, 72, or 96 h, cell viability was analyzed by microplate reader. The MTT results indicated that 3-DSC began to significantly inhibit skin cancer cell proliferation at the concentration of 10 and 20 μM in these four cell lines (Figure 3A). To provide additional evidence supporting the inhibitory potential of 3-DSC in skin cancer cells, we performed a soft agar assay using skin cancer cells (A431, SK-MEL-2, SK-MEL-28, and A375) to determine whether cell growth is affected upon treatment with 3-DSC (Figure 3B). Our results illustrated that 3-DSC was able to affect anchorage-independent cell growth in A431, SK-MEL-2, SK-MEL-28, and A375 cells starting at a concentration of 10 μM. EGF is known to activate MAPK/ERK signaling (Pan et al., 2018). Thus, we performed an anchorage-independent cell transformation assay to test whether treatment of 3-DSC could affect the colony formation potential of normal skin cells cultured in the presence of EGF. Our results indicated that 3-DSC was effective in significantly inhibiting EGF-induced cell transformation at a concentration of 5 μM (Figures 3C,D). To give more evidence of the role of TOPK in proliferation of skin cancer, we established TOPK knock-down skin cancer cells from the TOPK highly expressing SK-MEL-2 and A375 cell lines. The results indicated that TOPK knock-down inhibited anchorage-independent colonies growth of skin cancer cells. We confirmed that treatment of SK-MEL2 and A375 TOPK knock-down cells with 3-DSC failed to inhibit anchorage-independent cell growth (Figures 3E,F).

### 3-DSC Induced Skin Cancer Cell Cycle Arrest at G2/M Phase and Cell Apoptosis

**FIGURE 4:**
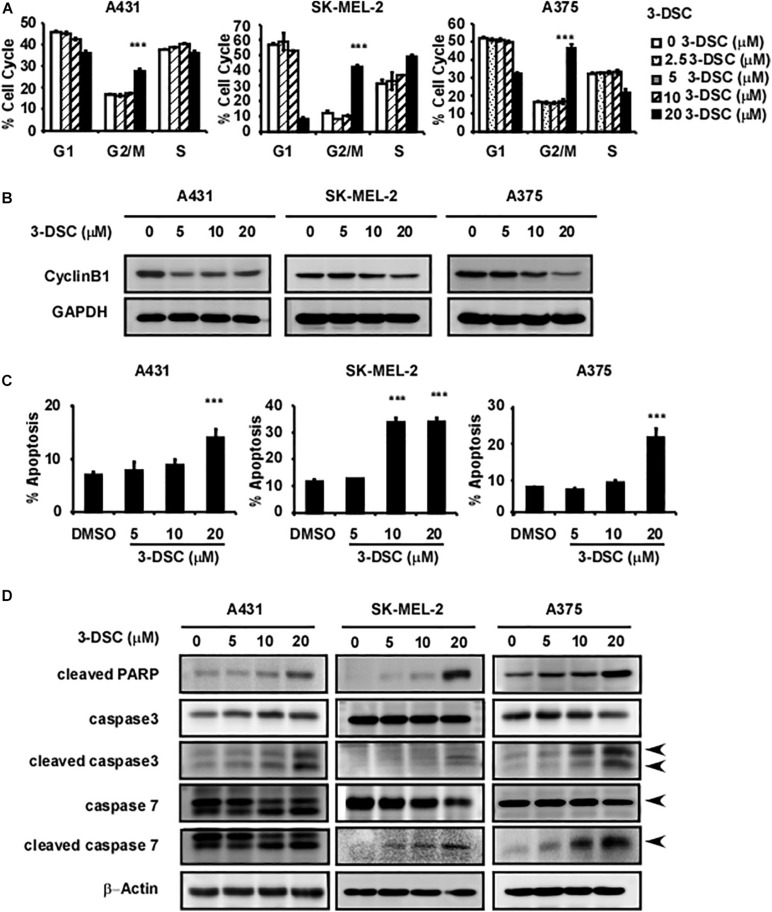
3-DSC induces arrest at G2/M phase and cell apoptosis in skin cancer cells. **(A)** Effect of 3-DSC to skin cancer cells’ cell cycle. **(B)** G2/M phase marker cyclin B1 protein expression level in skin cancer cell lines after treatment with 3-DSC at various concentrations. **(C)** 3-DSC induces apoptosis in skin cancer cell lines. Skin cancer cells were incubated with various concentrations of 3-DSC for 48 h and analyzed for apoptosis markers indicating pre/late apoptosis by flow cytometry. Data are shown as mean ± SD values. **(D)** Cell apoptosis marker cleaved PARP/cleaved caspase 3/cleaved caspase 7/PARP/caspase 3/caspase 7 expression after 3-DSC treatment. 3-DSC treatment increased cleaved PARP/cleaved caspase 3/cleaved caspase 7 protein expression in a dose-dependent manner. ***p < 0.001.

The results above suggest that 3-DSC treatment induces cell growth inhibition *in vitro*. However, the inhibition that we observed could be due to cell cycle perturbations, induction of cell apoptosis, or a combination of both. Thus, we first sought to determine whether 3-DSC treatment affects cell cycle dynamics in skin cancer cells. We applied 3-DSC to skin cancer cells before performing cell flow cytometry and western blotting using G2/M phase marker cyclin B1 (Figures 4A,B). The results indicated that 3-DSC induced cell cycle arrest at G2/M phase at a concentration of 20 μM. The western blot results verified that protein levels of the G2/M phase marker, cyclin B1, decreased at 20 μM. To assess whether 3-DSC contributes to cell death in addition to growth inhibition in skin cancer cells, we next performed a cell apoptosis assay. A431, SK-MEL-2, and A375 cells were incubated with or without 3-DSC for 24 h before being harvested and examined by cell flow cytometry. The results indicated a significant increase in the percentage of PI and annexin-V-positive cells (dead or apoptotic cells) in the 3-DSC-treated group relative to the untreated control cells (Figure 4C). Additionally, we detected apoptosis protein markers, cleaved PARP, cleaved caspase-3, cleaved caspase-7, caspase 3, and caspase 7 in 3-DSC-treated and untreated cells using western blot. The results showed that the cell apoptosis markers, cleaved-PARP, cleaved-caspase 3, and cleaved-caspase 7, were significantly increased upon treatment of skin cancer cells with 3-DSC in a dose-dependent manner (Figure 4D).

### 3-DSC Inhibited TOPK Signaling Pathway

**FIGURE 5:**
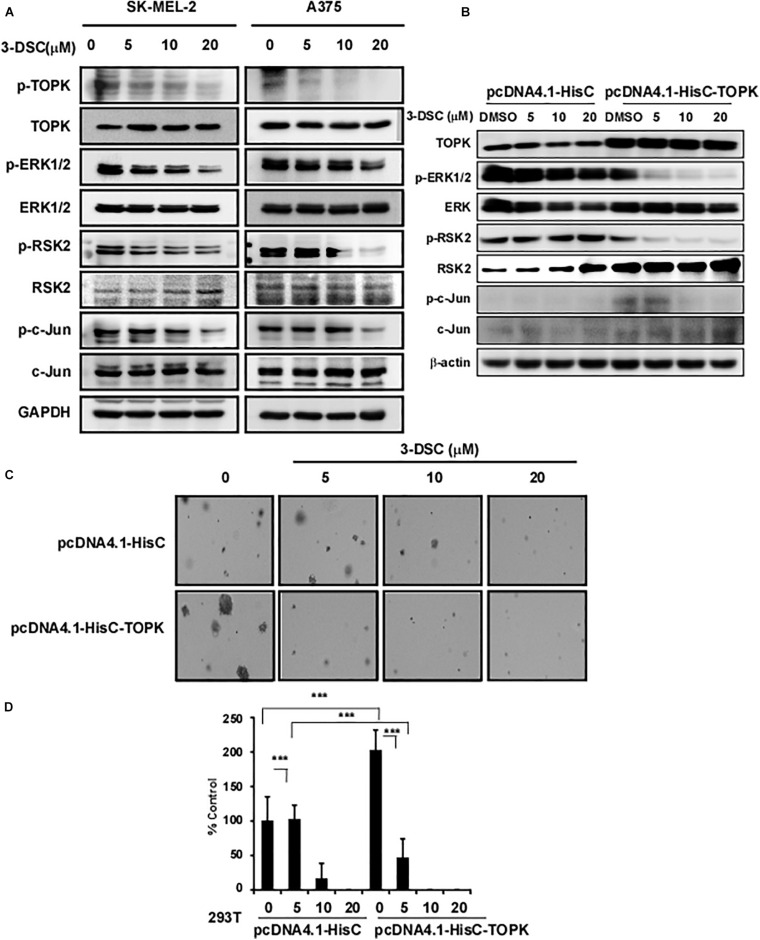
3-DSC inhibited TOPK signaling pathway in skin cancer cell lines. **(A)** p-TOPK, TOPK, and downstream effector protein expression after 3-DSC treatment. SK-MEL-2 and A375 cells were incubated with a culture medium with FBS and 3-DSC (5, 10, or 20 μM) or without 3-DSC for 2 h, and cell lysates were harvested before analysis by western blot. **(B)** TOPK was overexpressed in HEK-293T cells using pcDNA4.1-HisC (control) and pcDNA4.1-HisC-TOPK before cells were treated with or without 3-DSC at various concentrations. **(C)** Effect of 3-DSC on anchorage-independent growth in HEK-293T cells expressing *pcDNA4.1-HisC* and *pcDNA4.1-HisC-TOPK*. TOPK over-expression increased colony number and size. **(D)** Quantification of colony number in panel **(C)**. Data are shown as mean ± SD values. ***p < 0.001.

To determine the effect of 3-DSC on TOPK signaling in the context of skin cancer, we incubated SK-MEL2 and A375 cells over the concentration of 3-DSC ranging between 0 and 20 μM for 2 h before harvesting cell lysates to measure the protein level of TOPK and its downstream effectors by western blot. The results revealed that 3-DSC decreased the level of p-TOPK in a dose-dependent manner. Consequently, levels of downstream p-ERK, p-RSK, and p-c-Jun protein were also decreased due to attenuated p-TOPK (Figure 5A). To further confirm whether the inhibitory effect of 3-DSC is due to modulation of TOPK activity, we over-expressed TOPK in HEK-293T cell by transfection of the *pcDNA4.1HisC-TOPK* plasmid. The western blot results indicated that we successfully induced TOPK over-expression (Figure 5B). We then performed a soft agar assay using the HEK-293T cell line over-expressing TOPK in the presence or absence of 3-DSC. The results showed that over-expression of TOPK promoted proliferation in untreated HEK-293T cells; however, TOPK-induced proliferation of the HEK293T cells was reduced upon treatment with 3-DSC (Figures 5C,D). Thus, we concluded that 3-DSC inhibited skin cancer cell growth by reducing p-TOPK expression and the downstream effectors of TOPK.

### 3-DSC Inhibited Skin Cancer Cell-Derived Xenograft Growth

**FIGURE 6:**
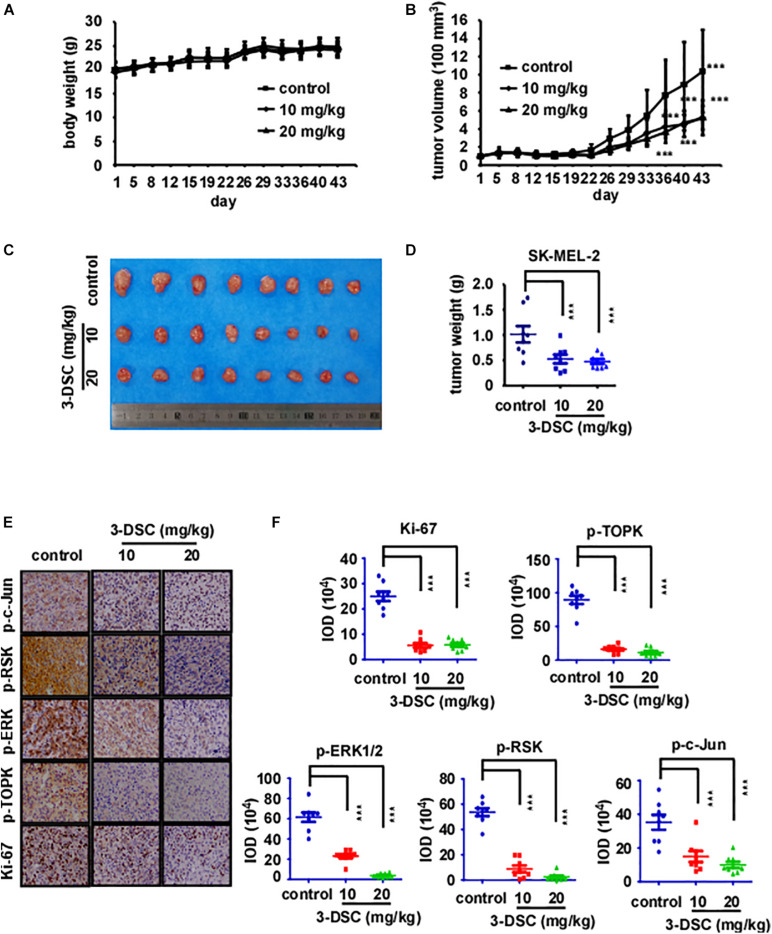
3-DSC inhibited skin cancer cell-derived xenograft (CDX) growth by attenuating TOPK expression. **(A)** Bodyweight of three groups of CDX mice over the duration of 43 days. Body weight was measured twice a week. **(B)** Tumor volume of three groups CDX mice over the duration of the experiment. Tumor size was measured twice a week. **(C)** Photograph showing tumors excised from control and 3-DSC-treated groups after euthanasia. **(D)** Quantification of tumor weight in panel **(C)**. Data are shown as mean ± SD values. **(E)** IHC results of CDX tumor tissues (Ki-67/p-TOPK/p-ERK/p-RSK/p-c-Jun) derived from control and 3-DSC-treated groups. **(F)** Quantification of IHC results shown in panel **(E)**. Data are shown as mean ± SD values. ***p < 0.001.

To determine the toxicity of 3-DSC *in vivo*, we treated Nu/Nu mice via abdominal injection with 3-DSC (20 or 40 mg/kg) or vehicle alone and measured body weight every day for 43 days. Mice were then euthanized and the liver and spleen were collected and weighed. The body, liver, and spleen weight indicated no significant toxicity of 3-DSC to mice at the administered concentrations (Supplementary Figures 2B,C). To determine whether the inhibitory effect of 3-DSC observed *in vitro* could be recapitulated *in vivo*, we applied 3-DSC to a CDX mice model. Mice were weighed (Figure 6A) and the tumor size (length × width × height × 0.52) was measured twice a week (Figure 6B). Results indicated that body weight steadily increased over the duration of the experiment. The tumor size indicated 3-DSC significantly reduced SK-MEL-2 CDX tumor growth (Figures 6C,D). We also performed IHC analysis on the tumor tissues to assess if the administered concentrations of 3-DSC were effective in attenuating TOPK signaling at the protein level *in vivo*. Indeed, the results indicated a significant decrease in the levels of p-TOPK, p-ERK, p-RSK, and p-c-Jun proteins (Figures 6E,F). Based upon our experimental results, we suggest that 3-DSC is able to prevent SUV damage to skin, inhibit skin cancer growth *in vitro* and *in vivo*, and induce apoptosis and cell cycle arrest at G2/M phase by attenuating TOPK activity through direct physical interaction.

## Discussion

Solar ultraviolet (sUV) irradiation as a major environmental carcinogen is the main risk factor that causes inflammation and skin cancer (Roh et al., 2018). Acute solar-simulated light was also found to rapidly activate PI3K/AKT/mTOR and MAPK signaling pathways in human tissues (Bermudez et al., 2015). As an anti-cancer target (Park et al., 2006; Alachkar et al., 2015; Ikeda et al., 2016; Ohashi et al., 2017) and upstream MAPK family member (Abe et al., 2000), TOPK may be potentially relevant for treating UV-induced skin cancer (Gao et al., 2017). TOPK and p-TOPK were highly expressed in skin cancer cell lines (Figure 1A) and in metastatic melanoma tumors compared with primary melanoma tumors according to GTEx and TCGA patient samples (Figure 1B). Independent cohort data presented within the Gene Expression Omnibus (GEO) database provide further evidence of heightened TOPK expression in cancer (Liu et al., 2019). In esophageal squamous cell carcinoma, TOPK was positively correlated with tumor metastasis (Seol et al., 2017; Zykova et al., 2017; Ma et al., 2019) by activating Src/GSK3β/STAT3 signaling pathway (Jiang et al., 2019). TOPK expression was also correlated with p53 expression (Hu et al., 2010; Lei et al., 2015) and cancer patients’ prognosis (Ikeda et al., 2016; Ohashi et al., 2016; Pirovano et al., 2017; Hayashi et al., 2018; Koh et al., 2018; Su et al., 2018; Xu and Xu, 2019; Zhang et al., 2019). These observations indicate that TOPK may show promise as a potential target for skin cancer treatment. Indeed, upon knocking down TOPK in SK-MEL-2 and A375 cell lines (Figures 3E,F), we showed that anchorage-dependent colony formation was inhibited.

According to our previous study, 3-DSC could bind with TOPK and inhibit colon cancer growth (Zhao et al., 2019). As targeting TOPK may be a strategy for the treatment of skin cancer, we performed experiments to verify the effect of 3-DSC on skin cancer. Kinase assay results showed a significant inhibitory effect of 3-DSC on TOPK kinase at 5 μM (Figures 1D,E) and the KD value of 3-DSC to TOPK was increased from 13.3 to 26.7 in the TOPK enzyme kinetics assay (Figure 1F). Moreover, the specific binding sites between 3-DSC and TOPK were Thr42 and Asn172 (Figures 1G–I), and these all suggested that the kinase activity of TOPK has been suppressed by 3-DSC (Figures 1A–I). 3-DSC showed a significant inhibitory effect in the cell proliferation of skin cancer cell lines (Figures 3A,B), which was also supported by cell cycle assay (Figures 4A,B) and apoptosis assay (Figures 4C,D). 3-DSC inhibited the phosphorylation of ERK/RSK/c-Jun, which are the downstream of TOPK (Figures 5A–D; Li et al., 2016).

Furthermore, targeting TOPK has been a strategy to prevent solar-related inflammation (Xue et al., 2017), and 3-DSC showed anti-inflammatory effects in previous research (Kim et al., 2014). In our study, we also found that 3-DSC prevented skin thickening, which was induced by acute SSL (Figure 2). Cell line−derived xenograft (CDX) models created by implanting cancer cell lines into immunodeficient mice have contributed largely to the development of cancer drug therapies (Lin et al., 2018). HI-TOPK-032, one of the TOPK inhibitors, was shown to inhibit tumor growth by nearly 50% in a HCT116 colon cancer cell-derived xenograft mouse model at a dose of 10 mg/kg (Kim et al., 2012). We are the first researchers to investigate the effect of 3-DSC *in vivo* by SK-MEL2 cell-derived xenograft mouse model. We confirmed its inhibitory effect via IHC, whereby we observed a significant reduction in Ki-67 and TOPK/ERK/RSK/c-Jun signaling protein expression (Figures 6E,F). Another TOPK inhibitor, acetylshikonin, was observed to inhibit tumor growth in colon cancer patient−derived xenograft (PDX) models compared with vehicle group at a dose of 120 mg/kg (Zhao et al., 2020). Whereas the inhibitory effect of 3-DSC to tumors is 50% with no significant changes to body, liver, and spleen weight, other TOPK inhibitors, such as OTS514 and OTS964, induced hematopoietic toxicity as reported in an *in vivo* study (Matsuo et al., 2014; Nakamura et al., 2015). The present study revealed that 3-DSC showed no obvious effect on the weight of liver and spleen.

In conclusion, as 3-DSC prevents SUV-induced damage, inhibits skin cancer *in vitro* and *in vivo*, and shows minimal side effects when administered at a high dosage, this compound could be considered a viable therapeutic option for the treatment of skin cancer and as a skin cancer therapeutic in further clinical research. The compound could potentially be used in pre-cancer stages or applied topically after extensive UV exposure with more efficiency and less side effect.

## Data Availability Statement

The original contributions presented in the study are included in the article/Supplementary Material, further inquiries can be directed to the corresponding author/s.

## Ethics Statement

The animal study was reviewed and approved by the Ethics Committee of China-US (Henan) Hormel Cancer Institute.

## Author Contributions

XF, RZ, and GY were involved in study concept and design, acquisition of data, analysis and interpretation of data, and drafting of the manuscript. XF, RZ, GY, FY, BX, and KVL performed the experiments. J-HS, KL, and BC provided material support. XF, RZ, M-HL, and ZD wrote the manuscript. ZD and M-HL had supervision of the whole study. All authors read and approved the final manuscript.

## Conflict of Interest

The authors declare that the research was conducted in the absence of any commercial or financial relationships that could be construed as a potential conflict of interest.

## Supplementary Material

Supplementary Figure 1 TOPK expression does not appreciably change across race, age, and stage. **(A)** RNA-seq data detailing TOPK transcript expression in tumors derived from Asian and Caucasian patients. **(B)** RNA-seq data detailing TOPK transcript expression in patient-derived tumor tissues was analyzed according to patient age. **(C)** RNA-seq data detailing TOPK transcript expression in patient-derived tumor tissues was analyzed according to tumor stage.

Supplementary Figure 2 3-DSC showed no toxicity in NFDH cells or mice. **(A)** Effect of 3-DSC in Normal Human Dermal Fibroblasts. NHDF cells were treated various concentrations of 3-DSC and cell growth was determined by MTT assay. **(B)** Influence of 3-DSC to mice body weight. Three mice per group were treated with 3-DSC (20 or 40 mg/kg) or vehicle solution and weighed once per day over 2 weeks. **(C)** Spleen and liver weight and photograph of control and 3-DSC-treated groups over the duration of 2 weeks for the toxicity experiment. No significance was observed between control and experiment groups. **(D)** Spleen and liver weight of control and 3-DSC-treated groups in 3-DSC CDX experiment. No significance was observed between control and experiment groups.
