# Supplementary figures and images for: 3-Deoxysappanchalcone Inhibits Skin Cancer Proliferation by Regulating T-Lymphokine-Activated Killer Cell-Originated Protein Kinase in vitro and in vivo
Source: Front Cell Dev Biol. 2021 Mar 25;9:638174. doi: 10.3389/fcell.2021.638174 (PMC8027363; doi:10.3389/fcell.2021.638174)

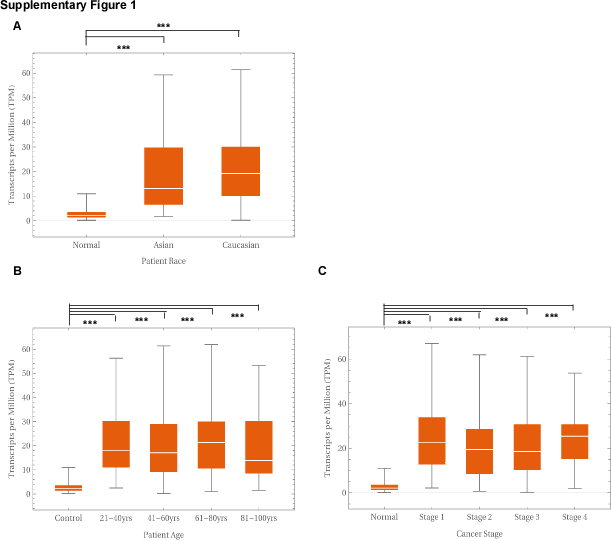

Supplement: Supplementary Figure 1 — TOPK expression does not appreciably change across race, age, and stage. (A) RNA-seq data detailing TOPK transcript expression in tumors derived from Asian and Caucasian patients. (B) RNA-seq data detailing TOPK transcript expression in patient-derived tumor tissues was analyzed according to patient age. (C) RNA-seq data detailing TOPK transcript expression in patient-derived tumor tissues was analyzed according to tumor stage. [file Image_1.TIF]

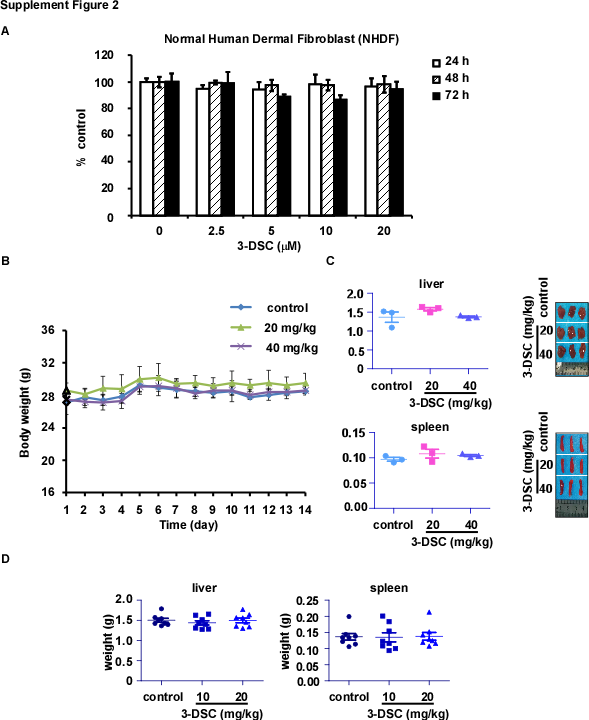

Supplement: Supplementary Figure 2 — 3-DSC showed no toxicity in NFDH cells or mice. (A) Effect of 3-DSC in Normal Human Dermal Fibroblasts. NHDF cells were treated various concentrations of 3-DSC and cell growth was determined by MTT assay. (B) Influence of 3-DSC to mice body weight. Three mice per group were treated with 3-DSC (20 or 40 mg/kg) or vehicle solution and weighed once per day over 2 weeks. (C) Spleen and liver weight and photograph of control and 3-DSC-treated groups over the duration of 2 weeks for the toxicity experiment. No significance was observed between control and experiment groups. (D) Spleen and liver weight of control and 3-DSC-treated groups in 3-DSC CDX experiment. No significance was observed between control and experiment groups. [file Image_2.TIF]
